# Molecular Mechanisms in Responses to Combined Stresses in Strawberry

**DOI:** 10.3390/cimb48080793

**Published:** 2026-08-05

**Authors:** Xiang Zhang, Xuemei Xia, Shuang Wang, Qi Sun, Lingxue Kong, Jiajie Yu, Xiaohong Li

**Affiliations:** School of Agriculture, Liaodong University, Dandong 118003, China; zhangxiang@liaodongu.edu.cn (X.Z.); 13624156318@163.com (X.X.); ws15542199225@163.com (S.W.); 15841132593@163.com (Q.S.); 18841513986@163.com (L.K.)

**Keywords:** strawberry, combined stress, signal transduction, gene regulation, transcription factors, stress resilience

## Abstract

Strawberry is a globally important yet stress-sensitive crop, increasingly threatened by combined abiotic and biotic stresses. Unlike single stresses, combined stresses elicit unique, non-additive responses through complex signaling and gene regulatory networks. This review synthesizes current knowledge on the molecular mechanisms underlying strawberry responses to combined stresses, focusing on signal perception and transduction as well as gene regulation. We examine how combined stresses are perceived by membrane-localized sensors and calcium channels, and how these signals are transduced through MAPK (mitogen-activated protein kinase) cascades, CDPKs (calcium-dependent protein kinases), and hormonal crosstalk involving ABA (abscisic acid), JA (jasmonic acid), and ethylene. At the gene regulation level, we discuss the roles of key transcription factors (WRKY, NAC (NAM, ATAF1, ATAF2 and CUC2), GRAS (GAI-RGA-and-SCR), DREB (Dehydration-Responsive Element-Binding protein), bZIP (basic leucine zipper transcription factor), CAMTA (calmodulin-binding transcription activator), ARF (auxin response factor), and LAV (Leafy Cotyledon2–Abscisic Acid Insensitive3–Val)), transcriptional cascades, epigenetic regulation via DNA methylation, and post-transcriptional (miRNAs such as Fan-miR73) and post-translational (ubiquitination and phosphorylation) control mechanisms. The review also evaluates emerging mitigation strategies informed by these molecular insights, including genomic selection, and explores future directions such as CRISPR (clustered regularly interspaced short palindromic repeats)-based genome editing and multi-omics integration. We conclude that understanding the integrated signaling and gene regulatory networks is essential for developing climate-resilient strawberry cultivars capable of withstanding increasingly complex stress combinations.

## 1. Introduction

Plants, as sessile organisms, are continuously exposed to a wide range of environmental challenges that threaten their growth, development, and survival. These stressors are broadly categorized into two types: abiotic stress, arising from non-living factors such as drought, abnormal light, salinity, and temperature extremes, and biotic stress, arising from living organisms such as fungi, bacteria, viruses, and insects [1,2,3]. The impact of stress to plants extends beyond individual plant growth, also profoundly affecting agriculture, ecology, biodiversity, and global food security [3,4,5]. In the context of climate change, the increasing incidence and intensity of stresses, including heat waves, prolonged droughts, floods, and storms, pose major threats to crop yield stability across all main agricultural regions [6]. According to the Intergovernmental Panel on Climate Change (IPCC), climate change-associated abiotic stresses increasingly cause massive crop losses worldwide [7], highlighting the urgent need for a deeper understanding of plant stress responses under accelerating climate change conditions.

In natural and agricultural contexts, plants rarely encounter a single stressor in isolation. Instead, they face combined or multifactorial stress conditions, such as drought with heat, salinity with drought, flooding with salinity, or any major abiotic stress combined with pathogen infection [8,9], referring to a series of scenarios in which plants encounter two or more stresses either simultaneously or sequentially [10]. In the conditions where three or more stresses co-occur, the term “multifactorial stress combination (MFSC)” is used to distinguish this more complicated situation from simple two-stress combinations [11]. A core and recognized principle is that the response to combined stresses is unique and cannot be directly predicted from a simple summation of the responses to individual stresses applied separately [12]. A recent meta-analysis assessing more than 120 published cases studying crop responses to combined heat and drought stress revealed that the combined stress caused, on average, twice the decrease in yield (relative to control) compared to exposure to heat stress alone [13]. The interaction between co-occurring stressors can be additive, synergistic (more severe than the summation of individual effects), or antagonistic (less severe) [14]. The mechanistic basis for these non-additive responses lies in the intricate and often conflicting signaling networks that govern plant stress adaptation. Different stresses activate distinct signaling pathways that interact or inhibit each other. For example, heat stress requires stomatal opening for transpirational cooling, whereas drought demands stomatal closure to conserve water—a physiological trade-off that makes combined heat–drought outcomes unpredictable from single-stressor studies [15]. Recent research has emphasized that understanding how plants integrate signals from multiple concurrent stressors, including reactive oxygen species (ROS), plant hormones (abscisic acid (ABA), jasmonic acid (JA), ethylene), calcium fluctuations, and chloroplast retrograde signaling, has emerged as a critical frontier in plant biology [16,17]. Furthermore, the impact caused by a certain stress combination is determined not only by the characteristics of the individual stresses, but also by their intensity, duration, occurrence order, plant developmental stage, and prior stress history [10,18].

The cultivated strawberry (mostly referring to *Fragaria* × *ananassa*) is a species of exceptional economic and nutritional importance. It is one of the most economically important berry fruits globally, valued for its attractive color, flavor, and high vitamin C content [19]. Despite its economic significance and widespread cultivation, strawberry is known to be stress-sensitive [20,21]. Optimal growing temperatures for strawberry plants range between 15 °C and 27 °C. Temperatures above 29 °C induce heat stress that reduces flower and fruit development, fruit set, and yield [22]. Temperatures above 30 °C significantly decrease leaf growth and photosynthesis, disrupt hormone regulation, and reduce fruit quality [23]. Low temperature stress can result in stunted growth, abnormal leaf and stem, delayed flowering, reduced metabolism and membrane fluidity, reduced fruit yield, and even demise [24]. Drought stress causes substantial reductions in fruit size, yield, and root development, as well as nutrient deficiencies [25]. Soil salinity disrupts osmotic balance and impairs physiological processes, further limiting productivity [26].

Strawberries are also highly susceptible to fungal diseases, which can be exacerbated by abiotic stresses. For instance, an elevated carbon dioxide (CO_2_) concentration of 780 ppm has been shown to disrupt salicylic acid (SA)–JA crosstalk, leading to a 73% increase in susceptibility to the gray mold *Botrytis cinerea* [27]. Under field conditions, strawberry plants frequently encounter simultaneous combinations of stresses: drought and heat, salinity and heat, or abiotic stresses together with pathogen pressure. Experimental evidence has shown that severe drought, soil salinization, and short extreme heat events all trigger acute injury effects on photosynthetic carboxylation capacity and photosystem II efficiency, with damage intensity varying by stress type [28]. Moreover, drought stress and high soil temperatures can synergistically increase root exudates, which facilitates pathogen colonization [29]. Recent research has highlighted that combined drought and salinity stress can reduce root biomass accumulation synergistically, with the magnitude of suppression exceeding that predicted from additive effects, and that osmotic adjustment, antioxidant defense activation, and hormonal crosstalk play critical roles in mediating these responses [11]. This scenario entails a serious risk for the future of strawberry production globally and calls for the development of new varieties with greater resilience to abiotic stresses [30].

Given the convergence of strawberry’s global economic importance, its high sensitivity to multiple stressors, and the increasing frequency and complexity of combined stress events under climate change, a comprehensive understanding of the molecular mechanisms by which strawberry plants perceive, integrate, and respond to co-occurring stresses is essential. While previous reviews on strawberry stress responses have predominantly focused on individual stresses or specific gene families, a comprehensive synthesis integrating the molecular mechanisms of signal perception, transduction, and gene regulation under combined stress conditions has been lacking. Furthermore, few studies have systematically examined how strawberry plants integrate conflicting stress signals through transcription factors or how epigenetic modifications mediate multigenerational stress memory. This review synthesizes current knowledge on the signal perception and transduction pathways as well as the gene regulatory networks underlying strawberry responses to combined abiotic and biotic stresses. Specifically, we examine how combined stresses are perceived by membrane-localized sensors and calcium channels, and how these signals are transduced through mitogen-activated protein kinase (MAPK) cascades, calcium-dependent protein kinases (CDPKs), and hormonal crosstalk involving ABA, JA, and ethylene. At the gene regulation level, we discuss the roles of key transcription factors (WRKY, NAC (NAM, ATAF1, ATAF2 and CUC2), GRAS (GAI-RGA-and-SCR), DREB (Dehydration-Responsive Element-Binding protein), bZIP (basic leucine zipper transcription factor), CAMTA (calmodulin-binding transcription activator), ARF (auxin response factor), LAV (Leafy Cotyledon2–Abscisic Acid Insensitive3–Val), transcriptional cascades, epigenetic regulation via DNA methylation, as well as post-transcriptional (miRNAs such as Fan-miR73) and post-translational (ubiquitination and phosphorylation) control mechanisms that collectively orchestrate stress adaptation. We also evaluate emerging mitigation strategies informed by these molecular insights, including genomic selection for resistance traits, and explore future directions such as clustered regularly interspaced short palindromic repeats (CRISPR)-based genome editing and multi-omics integration for developing climate-resilient strawberry cultivars.

By elucidating these mechanisms, this review aims to provide a theoretical foundation for breeding stress-resilient strawberry cultivars and developing sustainable cultivation practices capable of withstanding the multifaceted environmental challenges of a rapidly changing climate, thereby contributing to global food security and the sustainable development of the strawberry industry.

## 2. Stress Signal Perception and Transduction in Strawberry

The ability of strawberry plants to survive and acclimate to combined abiotic and biotic stresses depends critically on the rapid and accurate perception of stress signals and their efficient transduction into appropriate cellular responses. This section examines how strawberry plants perceive diverse stress signals and how these signals are transduced through interconnected networks.

### 2.1. Perception of Stresses

Temperature extremes, drought, and salinity are perceived through distinct but overlapping mechanisms. In plants, heat stress is sensed by plasma membrane fluidity changes and protein unfolding, which activate heat shock transcription factors (HSFs) and calcium channels [31,32]. In strawberry (*Fragaria vesca*), transcriptomic analyses have identified multiple heat-responsive sensor candidates, including membrane-associated receptor-like kinases (RLKs) and cyclic nucleotide-gated channels (CNGCs) [33,34]. Cold stress, on the other hand, is perceived by plasma membrane rigidification and calcium influx, leading to activation of the ICE1 (Inducer of CBF Expression 1)-*CBF* (*C-repeat binding factor*) transcriptional cascade [35,36,37]. Low temperature (LT) and low light (LI) often occur in conjunction. Strawberry (*F. ananassa*) plants perceive light signals through blue light receptor cryptochrome 1 (CRY1). The biological function of strawberry CRY1 in light signal transduction is proven by it promoting the expression of the downstream flowering-related gene. The expression of the strawberry *CRY1* gene is significantly induced by multiple abiotic stresses (low temperature, salt, and drought), which indicates the crosstalk between light perception pathways and stress signaling pathways.

Biotic stress perception in strawberry involves pattern recognition receptors (PRRs) that detect pathogen-associated molecular patterns (PAMPs) and damage-associated molecular patterns (DAMPs) [38,39]. In *F. vesca*, leucine-rich repeat receptor-like kinases (LRR-RLKs) have been systematically characterized, with subfamily XI-1 members showing high expression in leaves and roots, which suggests their role in pathogen detection [33]. Under combined abiotic and biotic stress, the simultaneous perception of PAMPs and abiotic signals creates a complex input that can lead to signal conflict or synergy. For instance, an elevated carbon dioxide (CO_2_) concentration of 780 ppm disrupts SA–JA crosstalk, increasing susceptibility to the gray mold *Botrytis cinerea* [27], indicating that the perception of one stress can alter the sensitivity to another.

### 2.2. Ca^2+^ Signaling

A near-universal response to both abiotic and biotic stresses is a rapid increase in cytosolic calcium concentration ([Ca^2+^]cyt) [40,41]. Calcium signals are generated by the opening of various Ca^2+^-permeable channels, including CNGCs, glutamate receptor-like channels (GLRs), and two-pore channels (TPCs) [42]. In strawberry (*F. ananassa*), annexin genes (*FaAnn5a*, *FaAnn5b*, *FaAnn8*) are involved in calcium-dependent stress responses [23]. These annexin proteins contain Ca^2+^-binding sites and peroxidase residues, suggesting their function as Ca^2+^-dependent regulators of ROS homeostasis. They also contain four annexin repeats and a GTP-binding motif, and their expression levels are differentially regulated by ABA, auxin, and calcium. This suggests a mediate crosstalk between hormone signaling and calcium signaling during strawberry development and stress responses. Under combined drought and heat stress, the amplitude and frequency of calcium oscillations differ from those induced by either stress alone, resulting in distinct downstream signaling outcomes [43].

### 2.3. Signal Transduction Cascades

Once perceived, stress signals are transmitted through a series of intracellular signaling cascades that amplify and diversify the initial input. Major transduction pathways in strawberry include MAPK cascades, CDPK pathways, and reactive oxygen species (ROS) signaling.

#### 2.3.1. MAPK Cascades

MAPK cascades are evolutionarily conserved signaling modules that transduce extracellular signals into intracellular responses [44,45]. A typical MAPK cascade consists of three sequentially activated kinases: MAPKKK, MAPKK, and MAPK [46]. In *F. vesca*, a comprehensive genome-wide analysis identified 12 *FvMAPK*, 7 *FvMAPKK*, and 73 *FvMAPKKK* genes [25]. Expression profiling revealed that *FvMAPK3* and *FvMAPK6* are induced 4- to 5-fold under salt and drought stress, while *FvMAPKK1* is upregulated 3- to 4-fold under cold stress, and *FvMAPKKK12* responds to heat stress (2- to 3-fold induction) [25]. Furthermore, the expression profiles of the *MAPK* and *MAPKK* genes in strawberry fruit and leaves have been investigated upon hormone and abiotic stress treatments, including salt, drought, high temperature, and low temperature, revealing that different stresses elicit distinct activation patterns of MAPK cascade components.

Under combined stress conditions, MAPK cascades exhibit non-additive activation patterns. For example, although direct evidence in strawberry is still lacking, studies in other plant species such as wheat and grapevine have demonstrated that simultaneous drought and heat stress can activate a different set of MAPKs compared with individual stresses [47,48]. Transcriptomic analysis has shown that MAPK cascades are significantly enriched pathways in strawberry stress responses and regulate downstream stress-responsive genes including DREB and COR family members [25,49]. The functional importance of MAPK signaling in strawberry stress tolerance is further supported by the observation that the overexpression of *RdreB1BI* (a DREB transcription factor) enhances drought tolerance through the activation of aquaporin-related genes [50].

#### 2.3.2. Calcium-Dependent and CDPK Signaling

The calcium signal is decoded primarily by calcium-binding proteins, including calmodulins (CaMs), CDPKs, and calcineurin B-like proteins (CBLs). In Rosaceae species, including strawberry, the CDPK gene family has been characterized. For instance, a genome-wide identification study in cultivated strawberry (*F*. *ananassa*) revealed nine *CDPK* genes that were differentially expressed in response to abiotic stress and hormone treatment [51]. Based on the conserved structural features of CDPKs, including a catalytic kinase domain and a calmodulin-like domain with EF-hand motifs for Ca^2+^ perception [52], these strawberry CDPKs are predicted to be activated upon Ca^2+^ binding and to phosphorylate diverse targets such as transcription factors, ion channels, and metabolic enzymes.

In strawberry (*F*. *ananassa*), the interplay between Ca^2+^ signaling and hormones is exemplified by the annexin genes. Exogenous ABA enhances the expression of *FaAnn5a* and *FaAnn8*, while auxin (IAA) promotes *FaAnn5b* expression [23]. Furthermore, calcium restrains the expressions of *FaAnn5s* (*FaAnn5a* and *FaAnn5b*) but promotes the expression of *FaAnn8* [23]. This complex regulation allows strawberry to fine-tune its responses according to the specific combination of stresses.

#### 2.3.3. Reactive Oxygen Species

While excessive ROS cause oxidative damage, controlled ROS production at sublethal levels serves as a critical signaling mechanism. Under combined stresses, ROS are generated by nicotinamide adenine dinucleotide phosphate (NADPH) oxidases (respiratory burst oxidase homologs, RBOHs), peroxidases, and organellar electron transport chains [53]. In strawberry, the expression of *FvRBOH* genes is induced by salt stress, with *FaRbohD* playing a key role in hydrogen peroxide (H_2_O_2_) accumulation and Na^+^ homeostasis [54]. Mechanistically, transcription factor FaWRKY40 directly binds to the *FaRbohD* promoter and activates its expression under salt stress, which leads to H_2_O_2_ accumulation that subsequently coordinates Na^+^ homeostasis through a reciprocal NO–H_2_O_2_ signaling circuit involving FaNR1. ROS signaling interacts closely with the Ca^2+^ and MAPK pathways: CDPKs (Ca^2+^-dependent protein kinases) are predicted to interact with RBOHs in strawberry [51], and ROS can activate Ca^2+^ signaling [55], while MAPKs such as FaMAPK5 and FaMAPK10 are involved in ABA-mediated H_2_O_2_ signaling [56], which suggests the existence of a self-amplifying signaling loop.

Under combined low temperature and low illumination (LTLI) stress, strawberry plants initially exhibit enhanced activities of superoxide dismutase (SOD), catalase (CAT), and peroxidase (POD). However, prolonged stress leads to decreased enzyme activities, accompanied by increased malondialdehyde (MDA) and H_2_O_2_ accumulation, which indicates antioxidant depletion and oxidative damage [57].

#### 2.3.4. Phytohormone

Phytohormones are core integrators that translate upstream signals into coordinated physiological and molecular responses [58]. Under combined stress conditions, the hormonal signaling network exhibits properties that cannot be predicted from individual stress studies.

ABA is the central regulator of osmotic stress responses, including drought and salinity [59]. In strawberry (*Fragaria nilgerrensis*), ABA biosynthesis genes, *NCED*, are rapidly upregulated under water deficit, leading to increased ABA accumulation [60,61]. ABA activates the PYL-PP2C-SnRK2 signaling module, which phosphorylates and activates downstream transcription factors such as ABA-responsive element-binding protein/ABRE-binding factor (AREB/ABF) and ion channels for stomatal closure [62,63,64]. JA and SA are key regulators of biotic stress responses, with JA typically controlling necrotrophic pathogens and herbivores, while SA is more involved in biotrophic pathogen defense [65,66,67]. In strawberry (*F*. *ananassa*), infection by the gray mold *Botrytis cinerea* (a necrotroph) triggers JA and ethylene signaling [65]. Under combined abiotic and biotic stress, complex crosstalk between the ABA and JA/SA pathways is frequently observed, which can be antagonistic or synergistic depending on the specific stress combination and its severity [68,69]. For instance, an elevated carbon dioxide (CO_2_) concentration of 780 ppm disrupts SA–JA crosstalk, leading to a 73% increase in strawberry (most possible cultivated strawberry *F.* × *ananassa*) susceptibility to the gray mold *Botrytis cinerea* [27]. In strawberry (*F.* × *ananassa*), exogenous application of JA has been shown to improve resistance to PEG-induced water stress by modulating the relative water content, photosynthetic pigments, and membrane stability index [68]. Ethylene, another phytohormone related to stress response, also participates in strawberry stress responses, with emerging evidence suggesting its dual roles in defense regulation and stress acclimation. Under combined low temperature and low illumination (LTLI) stress, strawberry (*F*. *ananassa*) plants exhibit enhanced oxidative stress and accelerated leaf senescence [57]. While direct evidence of ethylene biosynthesis gene induction under LTLI in strawberry is limited, studies in postharvest strawberry fruits have demonstrated that ethylene promotes ABA biosynthesis and interacts with ABA to regulate senescence [70,71,72], which suggests ethylene–ABA interactions under LTLI stress.

In summary, strawberry plants perceive combined stresses through diverse membrane-localized sensors and calcium channels, and transduce these signals through interconnected MAPK cascades, CDPK pathways, ROS signaling, and phytohormone networks. The non-additive nature of combined stress responses arises from the antagonistic and synergistic interactions at multiple nodes within these signaling pathways. Key components involved in these processes are summarized in Table 1.

## 3. Gene Regulation in Strawberry Response to Combined Stresses

The ability of strawberry plants to acclimate to combined stresses ultimately depends on the precise reprogramming of gene expression. This section examines the multi-layered regulatory networks, from transcription factors and transcriptional cascades to epigenetic modifications and post-transcriptional/translational controls, that govern strawberry’s gene expression under complex stress scenarios.

### 3.1. Transcription Factors as Core Regulators

#### 3.1.1. NAC Transcription Factors

The NAC family plays pivotal roles in plant development and stress responses [73,74]. A comprehensive genome-wide analysis in woodland strawberry (*F*. *vesca*) identified 37 *FvNAC* genes [26]. Expression profiling revealed that five *FvNAC* genes responded dramatically to various abiotic and biotic stresses, suggesting their contribution to stress resistance. Furthermore, *FvNAC* genes showed a greater response to cold treatment than to other abiotic stresses, and H_2_O_2_ elicited a stronger response than ABA, melatonin, and rapamycin. For biotic stresses, three *FvNAC* genes were upregulated during *Colletotrichum gloeosporioides* infection, while six were downregulated during *Ralstonia solanacearum* infection. Otherwise, the promoter activity of *FaNAC2* was induced by drought, salt, and cold stress, which demonstrates that a single NAC factor can integrate signals from multiple abiotic stress pathways [22]. Transgenic *Arabidopsis* overexpressing *FaNAC2* exhibited enhanced tolerance to salt and cold stress, with higher seed germination rates under simulated drought. Mechanistically, *FaNAC2* promotes proline biosynthesis genes while upregulating ABA biosynthesis genes, linking transcriptional regulation to osmotic adjustment and hormonal crosstalk. This multi-stress responsiveness suggests that *FaNAC2* may play a particularly important role under combined stress conditions.

#### 3.1.2. WRKY Transcription Factors

WRKY transcription factors are another major part of the participants in strawberry stress responses. A study on *F*. *vesca* identified 62 WRKY transcription factors (TFs) that responded to biotic (*Podosphaera aphanis*, powdery mildew) and abiotic (drought, salt, cold, heat) stresses [15]. Under powdery mildew infection, 33 *FvWRKY*s were upregulated and 12 downregulated, with *FvWRKY42* showing the highest induction. Drought and salt stress triggered greater *FvWRKY* upregulation than temperature stress, with *FvWRKY17* and *FvWRKY33* upregulated by 5- to 6-fold under drought [15]. Temperature stresses resulted in a greater downregulation of *FvWRKY* expression than drought and salt stresses. Moreover, the expression profiles of *FvWRKY* genes exhibited distinct patterns upon various hormones, including SA, MeJA, ethylene, and ABA [15]. This indicates the versatile roles of *FvWRKY* genes in strawberry plant responses to abiotic and biotic stresses.

Functional validation came from the overexpression of *FvWRKY42* in *Arabidopsis*, which enhanced powdery mildew resistance (40–45% fewer conidia), improved salt and drought tolerance (20–25% higher tolerance, 1.5-fold increased primary root length, 30–35% higher germination rate under stress), and increased ABA sensitivity (25–30% more stomatal closure) [75]. FvWRKY42 upregulated stress-related genes (*AtPR1*, *AtSOD*, *AtCAT*) by 3- to 4-fold. These results demonstrate that a single WRKY TF can integrate pathogen defense and abiotic stress responses. Given that *FvWRKY42* positively regulates both drought/salt tolerance and powdery mildew resistance, this gene may serve as a key integrator under combined abiotic and biotic stress scenarios, where plants often face simultaneous pathogen attack and environmental challenges.

#### 3.1.3. Other Emerging Transcription Factor Families/Subfamilies

Beyond the NAC and WRKY families discussed above, several other TF families, including GRAS, bZIP, DREB, C2H2 zinc finger proteins, CAMTA, ARF, and LAV, also contribute substantially to strawberry stress responses. In woodland strawberry, 54 *FveGRAS* genes were identified and divided into 14 subfamilies, with around half displaying increased or decreased expression under cold, heat, and gibberellic acid (GA_3_) treatments [76]. GRAS TFs are known regulators of gibberellin signaling and root development, and their stress responsiveness suggests roles in combined stress adaptation affecting root architecture [77,78]. A genome-wide analysis of bZIP family genes in woodland strawberry identified genes involved in drought and heat stresses [79]. bZIP TFs, particularly those of the ABI5 subfamily, are key downstream effectors of ABA signaling. In strawberry (*F*. *ananassa*), Fan-miR73 targets *ABI5*, with downregulation of the miRNA under stress leading to increased *ABI5* expression and enhanced stress tolerance [80].

DREB (dehydration-responsive element binding) TFs are central to cold and drought responses [81]. The isolation and expression analysis of *FaCBF1* from cultivated strawberry revealed its induction by low temperature and ABA [82]. Overexpression of *RdreB1BI* (a DREB homolog) in transgenic strawberry enhanced drought tolerance by activating aquaporin-related genes and increasing the relative water content [50]. The C2H2 zinc finger protein *FaZAT10* is implicated in responses to drought, salt, low temperature, ABA, and MeJA treatments [83], which suggests that it acts as a broad-spectrum stress regulator. CAMTA TFs decode Ca^2+^ signals and regulate cold-responsive *CBF* expression in *Arabidopsis* [84]. In strawberry (*F*. *ananassa*), the expression of all *FaCAMTA1*, *-3*, *-4*, and *-5* genes showed distinct alteration upon cold, heat, salt, and ethylene treatments [85]; ARF TFs play core roles in auxin signaling, which translates hormonal signals into the regulation of auxin-responsive genes [86]. In strawberry (*F. vesca*), *FvARF2* is responsive to exogenous IAA, which is a phytohormone proven to regulate plant stress response [87,88]; LAV TFs are proven to function in seed maturation and developmental stress responses. In strawberry (*F*. *ananassa*), *FaLEC2* directly regulates the transcriptional programming of LOX-derived volatile metabolism [89]. Key transcription factors and their functions are summarized in Table 2.

### 3.2. Transcriptional Regulatory Cascades and Co-Expression Networks

Transcription factors do not function in isolation; they form hierarchical regulatory cascades and co-expression networks that amplify and refine stress signals [90]. A powerful approach to dissect these networks is weighted gene co-expression network analysis (WGCNA). In drought stress treated *Fragaria nilgerrensis* (a wild strawberry relative with high drought resistance), WGCNA identified hub genes including *NCED* (*9-cis-epoxycarotenoid dioxygenase*, key for ABA biosynthesis), *CYP707A2* (*ABA 8′-hydroxylase*, involved in ABA catabolism), and *PP2Cs* (*protein phosphatase 2Cs*, negative regulators of ABA signaling) [61]. These hub genes are central to the ABA-dominated regulatory network and represent promising targets for genetic improvement.

In cold-stressed cultivated strawberry, transcriptomic analysis identified 2397 differentially expressed genes enriched in pathways including plant hormone signal transduction, flavonoid biosynthesis, MAPK signaling, starch and sucrose metabolism, circadian rhythm, and α-linolenic acid metabolism [91]. Notably, *GIGANTEA* (a circadian clock regulator), the two-component response regulator-like *PRR95*, and ethylene-responsive transcription factor *ERF105-like* were dramatically induced under low temperature, indicating that they function as key nodes in the cold regulatory cascade [91].

Under combined stresses, transcriptional cascades can exhibit emergent properties. For example, under combined drought and heat, the ABA-dependent cascade (NCED → ABA → PYL/PP2C → SnRK2 → AREB) is antagonized by heat-activated HSF-HSP cascades at multiple levels [48]. The net transcriptional output (e.g., the expression of *RD22*, *RD29*, or *HSP* genes) depends on the relative strength and timing of each stress input [92,93]. In strawberry, such antagonistic crosstalk has been inferred from physiological studies [94,95], but direct transcriptomic analysis under well-controlled combined stress conditions remains a priority for future research.

### 3.3. Epigenetic Regulation

In woodland strawberry, different stresses lead to substantial DNA methylation changes. Whole-genome bisulfite sequencing (WGBS) revealed that thermal stress causes genome-wide DNA methylation loss, with differentially methylated regions (DMRs) enriched near centromeric regions [19]. These DMRs were associated with stress-responsive TFs such as the *FvWRKY* and *FvNAC* genes, which suggests that DNA methylation directly regulates the expression of key transcription factors. Under heat, cold, drought, and salt stress, several DNA demethylase genes (e.g., *FvDME1*) were upregulated by 3- to 4-fold [16], which indicates active DNA demethylation as a response mechanism.

Furthermore, strawberry can transmit molecular stress memory over multiple asexual generations. Specific DNA methylation marks (epimutations) induced by heat stress are stably transmitted through stolons for at least three generations [34]. These epimutations are associated with persistent transcriptional changes in stress-responsive genes, which provides a heritable yet reversible form of adaptation.

### 3.4. Post-Transcriptional Regulation

**Figure 1:**
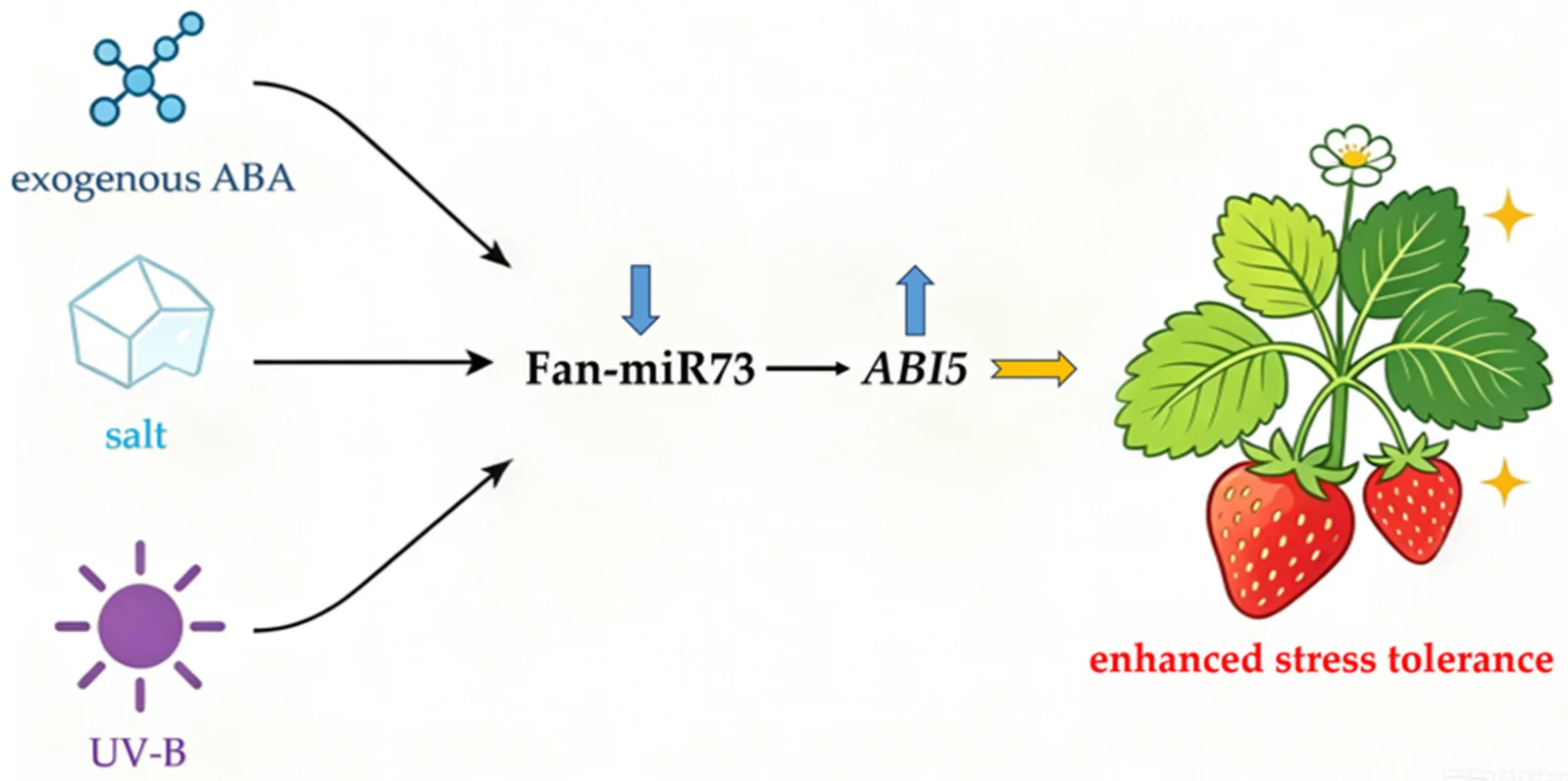
Post-transcriptional regulation of ABA signaling by Fan-miR73 targeting ABI5 in strawberry. Line arrows symbolize inducing. Thick solid arrows symbolize upregulation or downregulation. Dovetail-shaped solid arrow symbolizes leading to.

MicroRNAs are small non-coding RNAs that direct mRNA cleavage or translational repression [96]. In strawberry, a combined analysis of the sRNAome and transcriptome identified several differentially expressed miRNAs and their target genes, including transcription factors such as SPL (SQUAMOSA PROMOTER BINDING PROTEIN-LIKE), ARF (Auxin Response Factor), WRKY, bZIP, and TCP (TB1, CYC and PCF); these transcription factors are involved in fruit ripening and potentially in stress responses [97]. A well-characterized example is Fan-miR73, which targets the ABA signaling transcription factor *ABI5*. Under ABA treatment and stress conditions (salt and UV-B), Fan-miR73 expression is downregulated, leading to increased *ABI5* levels and enhanced stress tolerance [14]. This regulatory circuit (Figure 1) allows strawberry to rapidly amplify ABA signaling without altering *ABI5* transcription directly.

Other stress-responsive miRNAs in strawberry include those targeting ROS scavenging enzymes and ion transporters. For example, miR398 targets cytosolic superoxide dismutase (CSD) genes [98]; under stress, miR398 is downregulated, increasing CSD activity to control ROS levels.

### 3.5. Post-Translational Regulation

The ubiquitin–proteasome system (UPS) mediates targeted protein degradation, which allows for the rapid removal of negative regulators and turnover of signaling components [99]. In strawberry (*F*. *ananassa*), a genome-wide analysis identified *FaU-box* genes encoding E3 ubiquitin ligases, with expression profiling revealing roles in abiotic stress resistance and fruit ripening [100]. Specific members such as *FaU-box98* and *FaU-box136* show positive correlations with stress responses, while *FaU-box52* correlates with ripening.

Phosphorylation is the most widely studied PTM in stress signaling. MAPK cascades and CDPKs directly phosphorylate TFs, ion channels, and metabolic enzymes [101]. In strawberry annexins, Ca^2+^-dependent phosphorylation modulates their peroxidase activity and subcellular localization [23,102].

**Figure 2:**
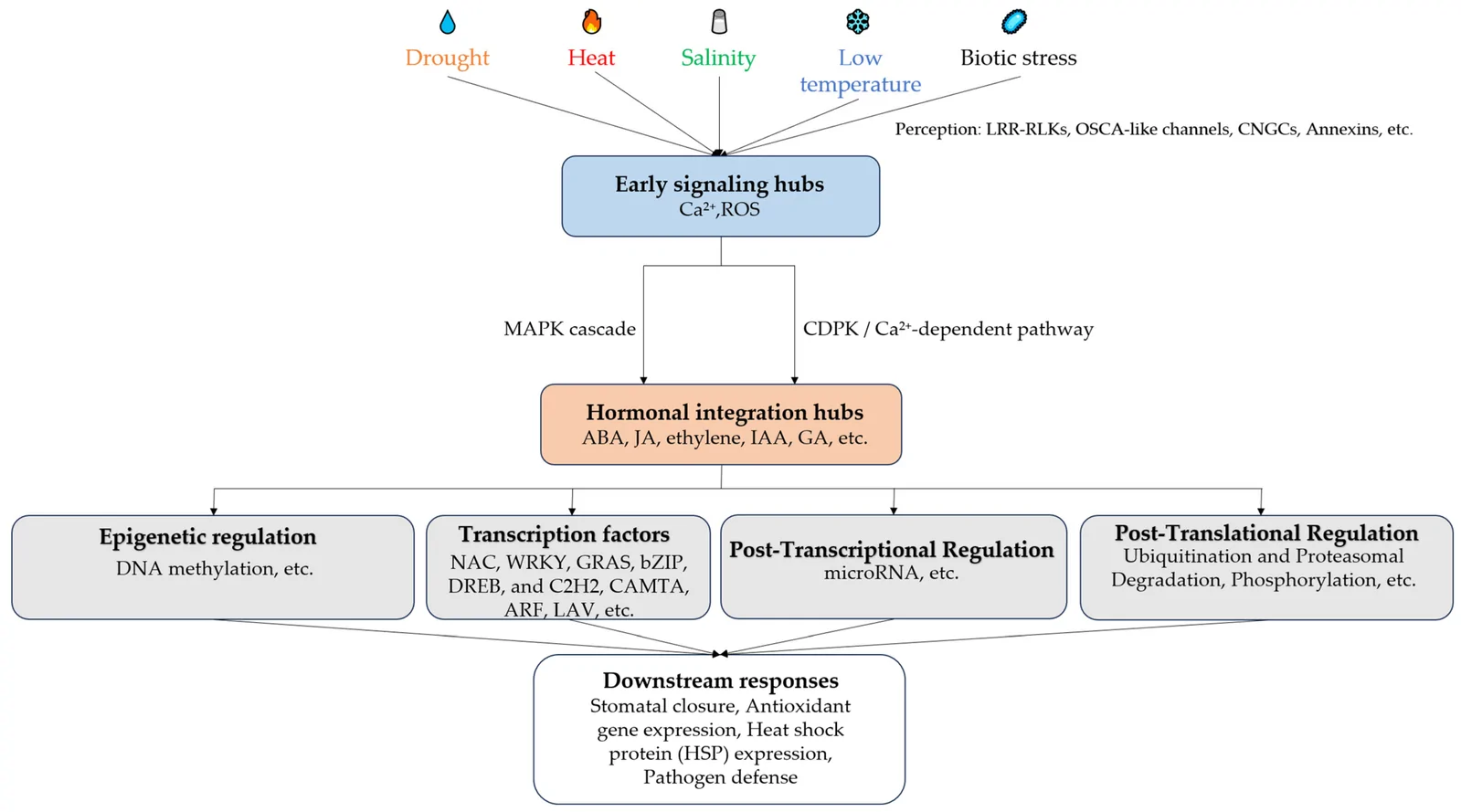
Multi-layered gene regulatory network in strawberry responding to combined stresses. This schematic illustrates the molecular pathway from stress perception and signal transduction to downstream gene expression.

In summary, strawberry plants employ a multi-layered gene regulatory system—spanning TF cascades, epigenetic memory, post-transcriptional control, and PTM networks—to cope with combined stresses (Figure 2). The non-additive nature of combined stress responses emerges from the complex interactions within and between these layers.

## 4. Discussion

The evidence synthesized in this review establishes that strawberry plants respond to combined stresses through complex, non-additive signaling and gene regulatory networks that cannot be predicted from individual stress studies. Unlike single stress conditions, combined stresses generate unique signal signatures, arising from the concurrent activation of often conflicting pathways that are integrated at multiple levels, from membrane-localized sensors to transcription factor cascades and epigenetic modifications.

It is important to note that the cultivated strawberry (*F.* × *ananassa*) is an allo-octoploid (2n = 8x = 56) formed by hybridization and polyploidization involving four diploid progenitor species [103]. In contrast, the woodland strawberry (*F. vesca*) is a diploid (2n = 2x = 14) with a small, well-annotated genome that serves as a valuable model system for studying gene function and stress responses [104]. The two species differ substantially in genome complexity and gene family size. For example, the PP2C gene family comprises 56 members in *F. vesca* but 228 in *F.* × *ananassa* [105], and the APX gene family includes 7 members in *F. vesca* versus 20 in *F.* × *ananassa* [106]. In octoploid strawberry, stress-responsive genes and regulatory elements have been preferentially retained in the dominant subgenome A, and subgenome dominance plays a major role in shaping stress response capacity [107]. Throughout this review, we distinguish findings from the diploid model *F. vesca* and the octoploid cultivated *F.* × *ananassa* where the original studies provide such information. However, it should be aware that direct functional validation in the octoploid background remains limited for many gene families, and findings from *F. vesca* may not always translate directly to cultivated strawberry. Furthermore, it should be acknowledged that direct experimental evidence on strawberry responses to specifically controlled combined stresses remains relatively limited compared to the extensive body of research on individual stresses. Part of the molecular mechanisms discussed in this review, including MAPK cascades, transcription factors from the WRKY and NAC families, calcium signaling, and DNA methylation, have been mainly characterized under single stress conditions. However, the emerging evidence from a growing number of studies indicates that the regulatory nodes identified under single stresses are also recruited under combined stress scenarios. For instance, in strawberry, the promoter activity of *FaNAC2* is induced by multiple individual stresses (drought, salt, and cold) [22], and *FvWRKY42* positively regulates both abiotic (drought, salt) and biotic (powdery mildew) tolerance [75]. It suggests that these hubs are likely to play integrative roles under combined stress conditions.

This discussion highlights the key principles emerging from the study of signal perception/transduction and gene regulation in strawberry under combined stresses, evaluates current knowledge gaps, and outlines future priorities for developing resilient cultivars.

### 4.1. Challenge of Signal Integration: Antagonism, Synergy, and the Competitive Transcription Factor Marketplace

A central insight from this review is that combined stresses force strawberry plants to resolve signaling conflicts. The classic antagonism between drought (ABA-driven stomatal closure) and heat (demand for stomatal opening) exemplifies this challenge [15,48]. At the molecular level, the resolution occurs through what has been termed a “competitive transcription factor marketplace”, where ABA, JA, GA, and ethylene signals converge on shared *cis*-elements and common transcription factor targets. In strawberry, the promoters of key stress-responsive genes such as *FaNAC2* and *FvWRKY42* contain multiple hormone-responsive elements (ABRE, JARE, GARE, ERE) [22,75], providing a physical basis for competitive integration.

The non-additive nature also extends to upstream signaling components. As illustrated in Section 2.3.1, MAPK cascades are key transducers of both abiotic and biotic signals, with different MAPK components showing distinct activation patterns under different individual stresses [25]. The critical question for combined stress responses is how these MAPK modules integrate convergent signals from the ABA, JA, and ethylene pathways. The observation that combined drought and heat stress in grapevine activates a set of MAPKs distinct from those activated by either stress alone suggests that signal competition at the MAPK level may represent a general principle of stress integration [47]. Direct evidence in strawberry, however, remains to be obtained. Similarly, calcium signaling, decoded by annexins and CDPKs, is modulated by both ABA and auxin in strawberry [23], and under combined stress, the interplay between Ca^2+^, ROS, and hormone signals becomes even more intricate.

A major knowledge gap remains: most studies to date have examined only one or two signaling components under single stresses. A systematic analysis of MAPK, CDPK, and hormone signaling under well-controlled combined stress conditions is urgently needed. Furthermore, the existence of stress-specific “signature” combinations (e.g., unique phosphopeptides or Ca^2+^ oscillations) has been proposed in model plants but not yet characterized in strawberry. High-throughput phosphoproteomics and calcium imaging in strawberry lines with biosensors would address this gap.

### 4.2. Transcriptional and Epigenetic Networks as Hubs for Stress Memory and Multigenerational Adaptation

Transcription factors of the NAC, WRKY, GRAS, and DREB families serve as master regulators that integrate upstream signals and orchestrate downstream gene expression. Among the transcription factor families discussed in Section 3.1, WRKY and NAC family members are particularly notable for their dual functionality. *FvWRKY42*, for example, positively regulates both abiotic and biotic stress responses [75]. This dual functionality is rare but highly valuable for combined stress scenarios where pathogens often attack plants already compromised by abiotic stress. The existence of such “multi-stress” TFs suggests that targeted overexpression or CRISPR activation of these genes could confer broad-spectrum resilience. Conversely, negative regulators like *FvWRKY53* (which represses flavonoid biosynthesis under boron excess) [108] are promising targets for knockout or suppression. While direct functional evidence for their roles in strawberry stress responses remains limited compared to model plants, their known functions in Ca^2+^ signal decoding (CAMTA), auxin-mediated stress adaptation (ARF), and developmental stress responses (LAV) suggest that they may represent important but currently underexplored components of strawberry’s stress regulatory network. The pathogenesis-related protein 10 (PR10) family, also known as Fra a 1 proteins in strawberry, represents a major group of defense-related proteins that are induced under both biotic and abiotic stress conditions. In *Fragaria vesca*, 28 *FvPR10* genes have been identified. Several *PR10* genes are induced upon infection by *Colletotrichum gloeosporioides* [109]. Four PR-10 proteins in strawberry leaves and 8 in strawberry roots are also highly induced in response to *Verticillium dahlia* [110]. These results indicate the important roles of *PR10* genes in strawberry’s defense against pathogens. Proline accumulation is a well-established adaptive mechanism for osmotic adjustment under water deficit and salinity stress. The key enzyme in proline biosynthesis, Δ^1^-pyrroline-5-carboxylate synthetase (P5CS), has been functionally characterized in strawberry [111]. Transformation of strawberry cultivars with the *P5CS* gene enhances drought tolerance by increasing proline accumulation, chlorophyll content, shoot growth, and biomass under drought-stress conditions. These findings highlight the importance of proline biosynthesis pathways in strawberry stress adaptation. While this review mainly discusses signaling and gene regulation, it is important to note that the downstream regulatory networks often involve the reprogramming of primary and secondary metabolism, an area that awaits future investigation in the context of combined stresses.

Epigenetic regulation adds a dynamic and heritable layer to gene control. In strawberry, heat stress induces genome-wide DNA hypomethylation, and specific differentially methylated regions (DMRs) are associated with stress-responsive TF genes [19,34]. Remarkably, some of these methylation changes are stably transmitted through at least three asexual generations, creating a form of stress memory [34]. This finding has profound implications for combined stress scenarios: a prior exposure to one stress (e.g., heat) could epigenetically prime the plant for a more robust response to a subsequent different stress (e.g., drought). However, direct evidence for cross-stress epigenetic priming in strawberry is lacking. Furthermore, whether combined stresses induce unique DMRs that are not observed under individual stresses remains unexplored.

Histone modifications and chromatin remodeling are likely involved but have received minimal attention in strawberry. In *Arabidopsis*, H3K4me3 and H3K9ac mark heat-inducible genes [112]; similar mechanisms are anticipated in strawberry but await experimental validation. The integration of DNA methylation, histone marks, and non-coding RNAs (e.g., Fan-miR73 targeting *ABI5* [14]) into a coherent regulatory network under combined stresses represents a frontier for future research.

### 4.3. Post-Transcriptional and Post-Translational Regulation: Rapid Adjustment and Signal Termination

While transcriptional reprogramming is essential for sustained adaptation, post-transcriptional (miRNA, alternative splicing) and post-translational (ubiquitination, phosphorylation) mechanisms enable rapid adjustments and signal termination. The Fan-miR73-*ABI5* module exemplifies miRNA-mediated control: under stress, downregulation of the miRNA leads to increased ABI5 protein without new transcription, accelerating ABA responses [14]. Under combined stresses where ABA signaling is antagonized (e.g., by heat), the regulation of Fan-miR73 itself may be modulated by additional signals—a hypothesis that can be tested using promoter-reporter constructs and stress combinations.

Alternative splicing (AS) is largely unexplored in strawberry stress responses. In other plants, the AS of *HSP* genes, *SR* splicing factors, and *RDC1*-like genes is induced by heat and cold [113,114,115]. Given that strawberry experiences both heat and cold stress depending on season and region, AS likely contributes to stress adaptation. Genome-wide AS analysis under combined stresses (e.g., cold + low light, drought + heat) would identify novel splice variants with potential regulatory functions.

At the protein level, ubiquitination by U-box E3 ligases controls the stability of key signaling components. In strawberry, *FaU-box98* and *FaU-box136* are induced by abiotic stress [100]; their targets are unknown but likely include PP2Cs (negative regulators of ABA signaling) and certain TFs. Under combined stress, the balance between ubiquitin-mediated degradation and protein synthesis determines the steady-state levels of signaling hubs. Phosphorylation by MAPKs and CDPKs adds another layer; for instance, annexin proteins are phosphorylated in a Ca^2+^-dependent manner, modulating their peroxidase activity [23]. The intersection of phosphorylation and ubiquitination (i.e., phospho-degrons) is a sophisticated regulatory mechanism that remains to be explored in strawberry.

### 4.4. Future Directions: From Mechanistic Understanding to Resilient Cultivars

The ultimate goal of studying signal perception and gene regulation is to develop strawberry cultivars that maintain yield and quality under combined stresses. Several promising avenues have emerged from the mechanistic insights discussed in this review.

First, transcription factor engineering. Overexpression of *FvWRKY42* in *Arabidopsis* enhanced both powdery mildew resistance and drought/salt tolerance [75]. Similar strategies in cultivated strawberry (using cultivar-specific promoters) could yield multi-stress tolerant lines. Conversely, CRISPR-Cas9 knockout of negative regulators such as *FvWRKY53* [108] may relieve the repression of flavonoid biosynthesis, enhancing antioxidant capacity under stress. The octoploid genome requires multiplex editing to target all homoeoalleles, but recent advances in CRISPR technology make this feasible.

Second, epigenetic breeding. The heritability of stress-induced DNA methylation marks through stolons [34] opens the possibility of selecting or generating epilines with enhanced stress memory. For example, exposing mother plants to repeated mild heat stress is promising to produce daughter plants with constitutively hypomethylated stress-responsive genes, leading to improved tolerance. This approach avoids transgenesis and could be rapidly deployed in commercial nurseries. However, the stability of epimarks under field conditions and their interactions with genetic variation need thorough investigation.

Third, multi-omics-guided precision breeding. Integrated transcriptomic, methylomic, and metabolomic analyses have already identified hub genes (e.g., *NCED*, *CYP707A2*, *PP2C* in ABA signaling [61]) and key pathways (flavonoid, phenylpropanoid, ABC transporters [116]). These datasets can be used to train genomic prediction models for combined stress tolerance, similar to the successful genomic selection for Verticillium wilt resistance [117]. By incorporating stress-specific expression quantitative trait loci (eQTLs) and methylation QTLs (mQTLs), breeders can select for alleles that confer the optimal regulatory responses under multifactorial stress.

Fourth, interdisciplinary field validation. Most mechanistic studies are conducted in controlled environments with single or double stresses. Field conditions involve fluctuating stresses (diurnal temperature cycles, intermittent drought, pest pressure). Collaborations between molecular biologists, physiologists, and agronomists are essential to translate laboratory findings into practical recommendations. For instance, the efficacy of AMF inoculation [6] or Se/SiO_2_ nanoparticles [14] under combined drought–heat–pathogen scenarios should be tested in multi-location field trials.

Overall, strawberry plants respond to combined stresses through a highly integrated signaling and gene regulatory network that exhibits non-additive properties. Key nodes in this network, including specific MAPKs, calcium sensors, transcription factors from the WRKY and NAC families, DNA methylation marks, and miRNA-target modules, have been identified. However, most studies remain reductionist and single-stress focused. Future research needs to systematically address stress combinations using multi-omics, genome editing, and field phenotyping to develop the resilient cultivars needed for sustainable strawberry production under climate change.

## 5. Conclusions

This review provides the first comprehensive framework that integrates signal perception, transduction, and gene regulation in strawberry under combined stresses. Strawberry plants respond to combined stresses through non-additive signaling and gene regulatory networks that integrate often conflicting inputs from the ABA, JA, ethylene, and heat stress pathways. Signal integration occurs at multiple levels—from calcium and MAPK cascades to a “competitive transcription factor marketplace” where distinct signals converge on shared transcription factors (WRKY, NAC, GRAS, DREB, CAMTA, ARF, and LAV) and *cis*-elements. FvWRKY42 and FaNAC2 exemplify hubs that coordinate abiotic and biotic tolerance, while epigenetic mechanisms, particularly DNA methylation, mediate stress memory transmissible over asexual generations. Post-transcriptional (miRNAs such as Fan-miR73) and post-translational (ubiquitination by U-box E3 ligases) controls enable rapid response adjustment. Future breeding should leverage CRISPR editing of hub transcription factors, epigenetic selection for stress-memory epialleles, and multi-omics-guided genomic selection to develop resilient strawberry cultivars capable of withstanding increasingly complex climate-driven stress combinations.

## Figures and Tables

**Table 1:** Major components involved in combined stress signal perception and transduction in strawberry.

Component Class	Examples	Stresses	Function	Reference(s)
Stress sensors	LRR-RLKs, CNGCs,	Heat, cold, osmotic	Perception of physical stress signals	[33,34]
Calcium signaling	Annexins (FaAnn5a/b/8), CDPKs	Drought, salt, ABA, IAA	Calcium binding and signal decoding	[23,51]
MAPK cascades	FvMAPK3/6, FvMAPKK1, FvMAPKKK12	Drought, salt, cold, heat	Signal amplification and transduction	[25]
ROS signaling	RBOHs, SOD, POD, CAT	Multiple stresses	Signaling and oxidative stress response	[53,57]
Hormone hubs	ABA (SnRK2, AREB), JA, SA, ethylene	Combined stresses	Integration of multiple stress signals	[64,65,68]

**Table 2:** Key transcription factors involved in strawberry stress response.

TF Family	Involved Stresses in Plants	Gene Name	Inducing Stress(es) in Strawberry	Target/Function in Strawberry	Reference(s)
NAC	Drought, salt, temperatures, flooding, oxidative, nutrition deficit, biotic	*FaNAC2*	Drought, salt, cold	Promotes proline biosynthesis; upregulates ABA biosynthesis genes; enhances stress tolerance in transgenic *Arabidopsis*	[22]
	Drought, salt, temperature extremes, nutrition deficit, biotic	*FvNAC* (multiple, 37 genes)	Cold, heat, drought, salt, H_2_O_2_, ABA, melatonin, rapamycin, biotic (*Colletotrichum*, *Ralstonia*)	Genome-wide identification; expression profiling under multiple stresses	[26]
WRKY		*FvWRKY42*	Powdery mildew, drought, salt	Upregulates *AtPR1*, *AtSOD*, *AtCAT*; enhances ABA sensitivity (stomatal closure); improves pathogen and osmotic stress resistance	[15]
		*FvWRKY17*, *FvWRKY33*	Drought, salt	Stress-responsive; potential integration with hormone signals	[15]
		*FvWRKY25*	ABA	ABA-regulated; role in stress signaling	[15]
GRAS	Drought, salt, temperature extremes, biotic	*FveGRAS* (54 genes, 14 subfamilies)	Cold, heat, GA_3_	Half of genes show altered expression under stress; roles in stolon development, fruit ripening, and stress adaptation	[76]
bZIP	Drought, salt, temperature extremes, biotic	Multiple (genome-wide identified)	Drought, heat	Stress-responsive	[79]
DREB	Drought, salt, cold, osmotic	*FaCBF1*	Low temperature, ABA	CRT/DRE-binding factor; cold acclimation	[82]
		*RdreB1BI*	Drought	Activates aquaporin genes (*FvPIP2;1*); increases relative water content; reduces electrolyte leakage	[50]
C2H2-ZFP	Drought, salt, temperature extremes, biotic	*FaZAT10*	Drought, salt, low temperature, ABA, MeJA	C1-2i subclass member; broad abiotic stress response	[83]
CAMTA	Cold, drought, salt, temperature extremes, biotic	*FaCAMTA1*, *-3*, *-4* and *-5*	cold, heat, salt and ethylene treatments	Stress-responsive	[85]
ARF	Drought, salt, temperature extremes, biotic	*FvARF2*	IAA treatment	Genome-wide identification; respond to exogenous auxin	[87]
LAV	Salt, drought, temperature extremes	*FaLEC2*	Hexanal content	Represses *FaLOX2* promoter during fruit ripening	[89]

## Data Availability

No new data were created or analyzed in this study. Data sharing is not applicable to this article.
